# Plate nail constructs for complex proximal tibia fractures

**DOI:** 10.1016/j.tcr.2025.101218

**Published:** 2025-07-10

**Authors:** Daniel Marks, Matthew Dulas, Solomon Egbe, James Dahm, Anthony Christiano, Jason Strelzow

**Affiliations:** a Department of Orthopaedic Surgery and Rehabilitation Medicine, The University of Chicago Medical Center, Chicago, IL 60637, USA; b The University of Chicago Pritzker School of Medicine, Chicago, IL 60637, USA; c The Indiana Hand to Shoulder Center, Indianapolis, IN 46260, USA; d Washington University of St. Louis, Department of Orthopaedic Surgery, St. Louis, MO 63110, USA

**Keywords:** Case series, Fracture, Proximal tibia, Intramedullary nailing, Plating

## Abstract

**Purpose:**

To report upon a series of patients who underwent a combined minimally invasive plate osteosynthesis and intramedullary nailing surgical approach for AO/OTA 41C2/3 +/− 42, 41B2/3 + 42 fracture types and highlight the surgical methodology for application.

**Patients:**

15 patients were treated with combined plate and intramedullary nail constructs at an academic urban trauma center from 2018 to 2022. All patients had AO/OTA 41C2/3 +/− 42 or 41B2/3 + 42 fractures.

**Intervention:**

The study intervention included retrospective review of patient charts and radiographs.

**Main outcome measures:**

Outcome measures included coronal and sagittal alignment at latest follow-up, intra-operative subsidence of articular fragments, reoperation, and complications such as infection, compartment syndrome, screw migration, or component failure.

**Results:**

The average follow-up for patients included was 6.2 months. At final follow-up, 13 patients had available post-operative assessments for review. At latest follow-up, the average coronal alignment ranged from 3.1 degrees of varus to 2.3 degrees of valgus, average sagittal alignment from 2.6 degrees of recurvatum to 2.0 degrees of procurvatum. There was no evidence of intra-operative or post-operative radiographic subsidence of the plateau. No patients underwent reoperation. There was one case of superficial infection and one case of proximal screw loosening.

**Discussion:**

Plate and nail constructs are a practical option for complex intra-articular fractures of the proximal tibia with metaphyseal or diaphyseal extension (AO/OTA 41C2/3 +/− 42, 41B2/3 + 42). This series demonstrates acceptable radiographic alignment and good clinical results associated with these fracture patterns, with short-to-medium-term follow-up and an overall low complication rate.

## Introduction

Intra-articular fractures of the proximal third of the tibia account for approximately 5 % to 11 % of all tibia fractures [1,2]. Due to involvement of the articular surface, patients risk impaired knee function, persistent instability, and early-onset post-traumatic osteoarthritis (PTOA) [[3], [4], [5]]. Fractures of the proximal tibia also have higher complication rates, such as malunion, compartment syndrome, and vascular injury, compared to fractures of the diaphysis and distal tibia [6,7]. Additionally, due to the surrounding anatomy and the often traumatic, high-energy etiology of proximal tibia fractures, these injuries have the potential to produce complex injury patterns with significant damage to the surrounding soft-tissue envelope and present with metaphyseal extension and concurrent diaphyseal fractures [6,[8], [9], [10], [11]].

Treatment for tibial plateau fractures with ipsilateral metaphyseal or diaphyseal fracture components is challenging and complex. Surgical goals involve anatomic reduction and fixation of the articular surface and the re-establishment of native knee alignment to allow for early range of motion [1,4]. High-energy proximal tibia fractures can be treated stepwise, with initial stabilization via external fixation followed by delayed definitive fixation with open reduction and internal fixation to allow for appropriate soft tissue consolidation [4,10,11]. Currently, there is no consensus in the literature regarding the superior technique for managing complex intra-articular proximal tibia fractures involving the metaphysis or diaphysis. As such, treatment selection often depends on surgeon preference and surgical planning based on specific fracture characteristics [1,8].

There has been a growing interest in combining the implementation of peri-articular tibial plating and intramedullary nailing (IMN) in complex proximal tibial fractures. Dunbar et al. introduced a combined nail-plate technique by utilizing a unicortical plate to temporarily maintain reduction for IMN in Gustilo-Anderson type III open tibial fractures [12]. More recently, studies have also described nail-plate combination techniques being successfully utilized for proximal tibial shaft fractures [11,13,14]. Wright et al. described a technique for the combined use of a periarticular proximal tibia plate and nail for bicondylar tibial plateau fractures with a lateral approach for plate osteosynthesis with subsequent intramedullary nail passage [10]. As was hypothesized in nail-plate combinations in distal femur fractures, it is thought that combining IMN and plate fixation allows for more balanced distribution of forces between the bone and implants that allow for early return to weightbearing as the IMN acts to maintain the native alignment and resist varus or valgus deformity, while the plate provides reduction and fixation of the articular surface [1,10,15].

**Fig. 1:**
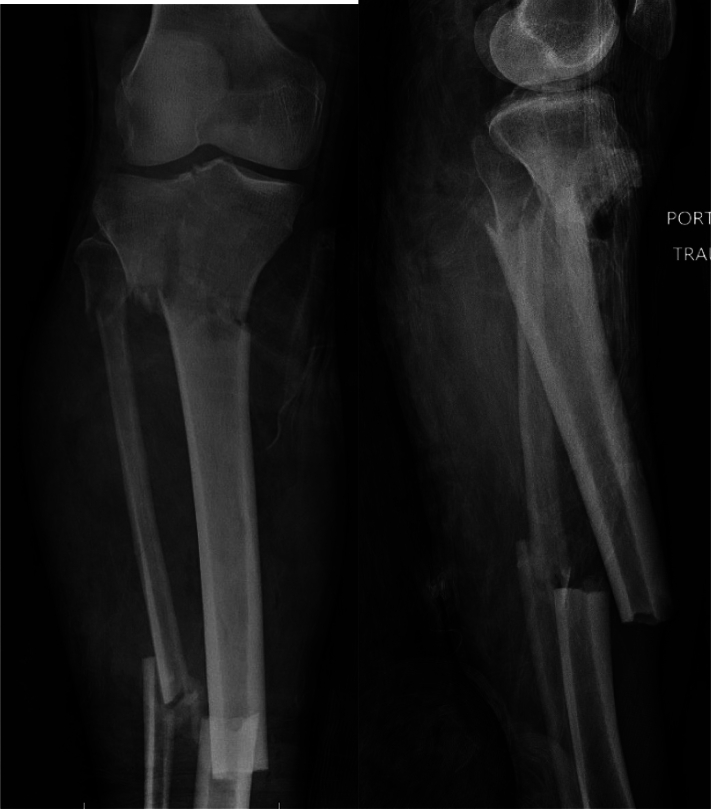
AP and lateral radiographs of a 54-year-old male (Case 13) involved in a building collapse/crush injury demonstrating 41C3/42A3 tibia fracture with multi-articular split-depression proximal tibia fracture with metaphyseal comminution, and concomitant ipsilateral transverse tibial shaft fracture.

The combined approach to complex fractures (AO/OTA 41C2/3 +/− 42, 41B2/3 + 42) may provide enhanced stability and alignment, allow for earlier return to weightbearing, and potentially less soft tissue insult via percutaneous insertion of an intramedullary nail. The study describes the surgical technique and experience of 15 patients who underwent combined plate-nail constructs for complex intra-articular proximal tibia fractures with ipsilateral metaphyseal or diaphyseal components at a single, urban level one trauma center (Fig. 1).

## Methods

### Patients

A retrospective review was performed of patients undergoing proximal tibia fixation at a single academic institution from 2018 to 2021. Inclusion for the series involved patients who received both open reduction internal fixation of intra-articular proximal tibia fractures and intramedullary nailing. Patients with extra-articular (AO/OTA 41A) proximal tibia fractures or fractures treated with mini-fragment plates for provisional reduction were excluded from the review.

A total of 15 patients met inclusion criteria, with 8 out of 15 (53 %) undergoing definitive management on hospital day 1, while 7 out of 15 (47 %) first underwent external fixation before definitive management on a later hospital day. Of the 15 patients included in the series, 13 were available for postoperative follow-up and clinical outcomes, with 2 lost to follow-up. Among these 13 patients, only 11 had radiographic follow-up beyond 3 months and were therefore included in radiographic bony union. Cases 4 and 8 were excluded from radiographic union assessment.

### Surgical technique

#### Pre-op

Pre-operative evaluation of the patients begins with standard history and physical exam with AP and lateral radiographs of the affected extremity. Particular attention is paid to the soft tissues as this often dictates timing for definitive surgery. Depending on fracture morphology and soft tissue status, the decision is made for provisional fixation with an external fixator or definitive fixation via combined plating with an intramedullary nail. CT imaging is typically obtained at the time of injury or following external fixation.

**Fig. 2:**
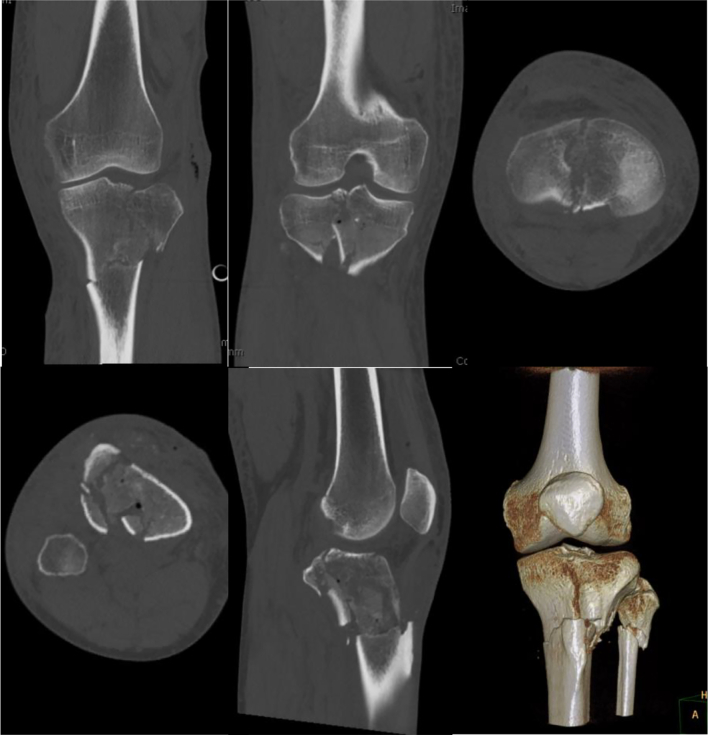
Coronal, axial, sagittal, and 3D reconstruction computed tomography images of Case 13, a 41C3/42A3 proximal tibial fracture component.

Several factors were considered regarding the combined plate-nail technique. Firstly, the soft tissue envelope is evaluated. Larger incisions required for plate fixation may not be amenable in some injuries, especially those with significant swelling and extensive soft tissue injury with or without internal degloving. This consideration is especially apparent if a combined dual-plate approach or a longer buttress plate is required. Secondly, radiographic assessment was performed on AP and Lateral views. Fractures with the following patterns are considered strong candidates for combined plate-nail surgical technique: 1) simple articular patterns and associated metaphyseal or shaft comminution and 2) ipsilateral split depression and distal shaft fractures (segmental). Simple intra-articular pathology that can be managed with single plating are ideal for this technique, however, dual-plating of the proximal fracture component can be performed as well. Next, CT was performed after external fixation or prior to single-stage open reduction internal fixation. CT imaging can be useful in determining the degree of comminution, aiding in the reduction of the articular surface, and assessing the ideal start point for an intramedullary nail. The identification of central and anterior comminution for the IMN has been considered an indication by some for avoidance of nail insertion due to the loss of proximal fixation and nail migration. This should be examined for on CT scans (Fig. 2).

#### Surgical technique

**Fig. 3:**
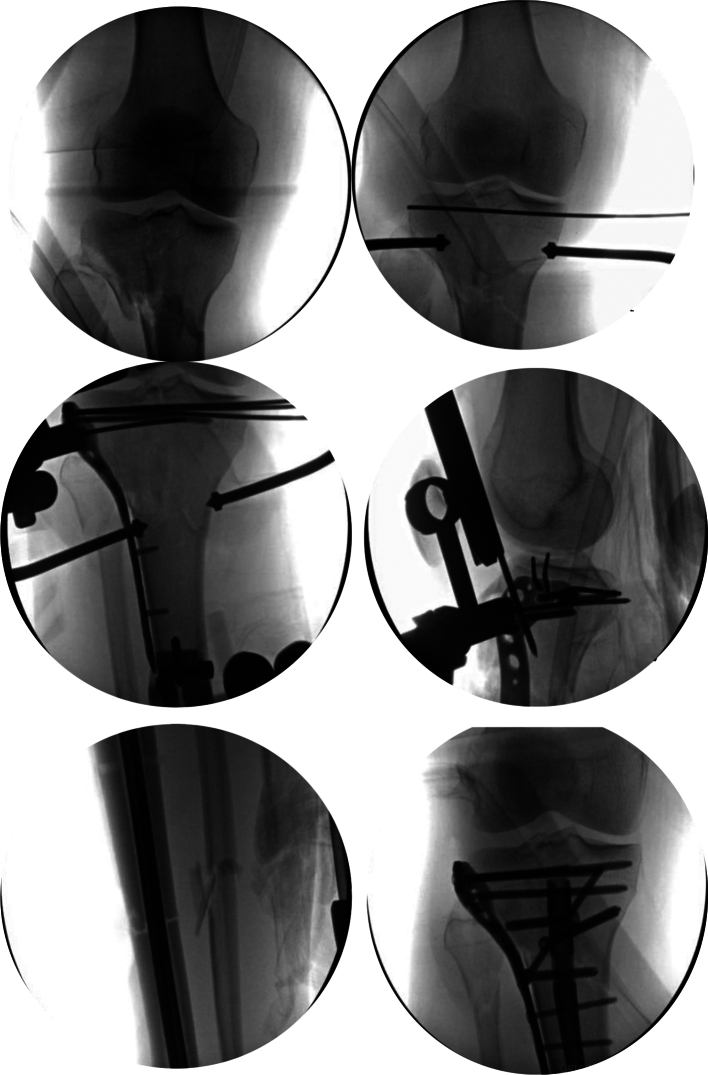
Intraoperative images of Case 13 demonstrating reduction of the articular surface and subchondral wiring, plate placement and preliminary fixation of 41C3/42A3 proximal tibia fracture with lateral plate, passage of intramedullary nail with conversion of unicortical to bicortical screws distally.

For surgical fixation, general anesthesia with skeletal relaxation was used in all cases. The patient was positioned supine on a radiolucent table. Pre-operatively, patients did not undergo a regional anesthesia block to allow post-operative neurological status assessment. All patients received pre-incisional antibiotics in accordance with institutional protocols. A tourniquet was applied to the affected extremity to be used, if necessary, during fixation of the tibial plateau and released prior to instrumentation for the intramedullary nail. The extremity was prepped and draped in sterile fashion. External fixator devices could be prepped into the field if desired, as demonstrated by recent studies, which showed no increase in infection rates with an external fixator prepped into the surgical field in patients with tibial plateau fractures [16,17]. Irrigation and debridement were performed in the open fracture setting as needed (Fig. 3).

A minimally invasive plate osteosynthesis (MIPO) technique was utilized for the approach to the proximal tibia. This technique, which involves dissection through operative windows and the use of outrigger assistance for screw insertion and percutaneous implant placement, has been proven effective in preserving fracture site biology for improved healing compared to open techniques [18,19]. A curvilinear incision was made from the lateral femoral epicondyle to the tibial tubercle adjacent to the tibial crest. The anterior compartment musculature was elevated off the tibia, including the subcutaneous layers and skin. This exposure was repeated proximally after the fascia lata was identified. Care was taken to preserve the joint capsule and lateral collateral ligament. Once satisfactory soft tissue exposure was achieved, reduction was performed typically with rotation and elevation of the articular fragments using point-to-point clamp with direct visualization via submeniscal arthrotomy or under fluoroscopic guidance. A large percutaneous clamp was then placed across the articular block before placement of K-wires in a subarticular position to hold the reduction. A periarticular plate was then selected and placed as close to the articular surface as possible. Freehand articular screws could be placed out of the trajectory of the IMN device as adjunctive fixation for the plate or in isolation if a plate is not felt to be necessary. Plate fixation positioning should ensure the reduction and compression of the articular fragments and provide support for any metaphyseal fragments. The periarticular plate should be placed as posteriorly as possible to allow unhindered passage of the intramedullary components. Selection of a plate large enough to comfortably span any metaphyseal comminution was recommended. Fixation began with the use of non-locking screws to secure the plate to the bone. These screws provided significant compression to both the fragments and the articular surface. This functioned to reduce the proximal tibial width and secured the articular block. Uni-cortical shaft screws were then placed distally through the plate to avoid blocking the nail. These unicortical screws could be swapped out later for bi-cortical screws following successful nail passage. Additionally, whirlybird positioners or clamps could be used instead of screws to avoid the need for replacing shaft screw fixation. Proximal screws, typically bi-cortical non-locking, were then added to the plate to act as raft screws and provide additional compression.

Next, attention was turned to the intramedullary nail. A trans-quadriceps, suprapatellar approach was preferred for all patients, optimizing incision burden by avoiding parallel incisions and maximizing the distance between incisions, thereby decreasing the likelihood of procurvatum deformity, which has been correlated with improved patient-reported outcomes and reduced intraoperative fluoroscopy time [20]. Moreover, the recent INSURT study demonstrated suprapatellar nailing to have significantly less knee pain at time points up to 1-year, increased weightbearing during kneeling, and higher rates of anatomic fracture reduction compared to infrapatellar nailing [21]. This approach was also helpful to avoid additional soft tissue injury and devascularization around the fracture site [20]. A lateral parapatellar approach could also be achieved by extending the incision from the tibial plate or making a separate incision lateral to the patella. The guide pin was placed just medial to the lateral tibial spine and anterior to the anterior margin of the articular surface of the tibial plateau. The guide pin was then advanced once satisfactory trajectory was confirmed on AP and lateral radiographs. With the aid of a soft tissue protector, the guide pin was over-drilled with an opening reamer to open the tibial canal. Special attention was made to ensure the soft tissue patellar guide was held firmly against the proximal tibia when reaming to protect articular cartilage. A ball-tipped guide wire was then passed through the opening but was not passed through the fracture site. Throughout all interventions near the articular surface, radiographic evaluation for subsidence was made. Reduction of any meta-diaphyseal or diaphyseal fracture was then performed typically with traction, manual manipulation, or reduction clamps. Once satisfactory reduction, overall alignment, and rotation were confirmed radiographically and clinically, the guidewire was passed until it was center-center above the plafond. After measurements were taken, sequential reaming was performed in standard fashion. The nail was then carefully passed. At least two proximal interlocking screws and at least one statically-locked distal interlocking screw were placed. Final radiographs were taken, and closure was performed based on surgeon preference. The patient was then placed in a soft dressing. Post-operatively, patients receive antibiotics for 24 h and venous thromboembolism chemoprophylaxis according to institutional protocol. Usually, immediate range of motion as tolerated and initial toe-touch weight bearing is recommended, with progressive return to full weight bearing by 6–8 weeks.

### Methods of assessment

Outcome measures assessed were alignment in both coronal and sagittal planes at follow-up, whether subsidence of articular fragments occurred intra-operatively, reoperation, and complications such as infection, compartment syndrome, screw migration, or component failure. Radiographic assessment was performed on most recent AP and lateral clinic x-rays. Coronal and sagittal plane alignment was measured by a senior resident. Other outcomes assessed were time to ambulation, knee range of motion at most recent follow-up, and weightbearing restrictions after surgery. Radiographic union was determined by bridging callus of 3 out of 4 cortices seen on AP and lateral X-rays.

## Results

A total of 15 patients underwent the above procedure, which included 12 males and 3 females. A total of 13 patients were included in post-operative analysis as 2 patients were loss to follow-up. Average age was 49.5 years and average BMI was 25.4. Five patients were active smokers at the time of injury. There were 7 open fractures. Mechanism of injury included 13 blunt injuries and 2 ballistic fractures. Six fractures were treated in a staged manner with external fixation before definitive treatment (Table 1). The remainder of the patients were treated in long leg splints or knee immobilizers before definitive treatment. One patient underwent four-compartment fasciotomies performed by the general surgery trauma service for concomitant vascular injury prior to definitive fixation. Average time until definitive fixation from injury was 4.7 days. Average time for follow up was 6.2 months. All patients were toe-touch weightbearing on the operative extremity with progression to full weightbearing by an average of 8.5 weeks except for one patient who was delayed to full weight bearing until 12 weeks, and 2 patients were made immediately full weight bearing post-operatively. Knee range of motion included an extension average of 2.2 degrees (range 0–7 degrees) and flexion average of 100.4 degrees (range 50–130 degrees) at 9.5 weeks postoperatively. Only 11 out of the 13 patients had at least 3 months of radiographic follow-up. All 11 of these patients demonstrated evidence of radiographic bony union. No knee instability was detected in any knees.

**Table 1:** Fracture classification and baseline characteristics of study population.

*N* = 15	
Average age (years)	49.5
Male	12 (80.0 %)
Female	3 (20.0 %)
AO/OTA Fracture Classification (Gustilo-Anderson Class)	
Case 01	41B3/42A1
Case 02	41B3/42A3 (GA2)
Case 03	41B3/42C2 (GA3B)
Case 04	41B3/42C3
Case 05	41C2/42A1
Case 06	41C2/42A3 (GA3A)
Case 07	41C2/42C2
Case 08	41C2/42C3 (GA3A)
Case 09	41C3/42A2 (GA3B)
Case 10	41C3/42A1 (GA1)
Case 11	41C3/42A1
Case 12	41C3/42A1
Case 13	41C3/42A3 (GA3A)
Case 14	41C3/42B2
Case 15	41C3/42C2
Blunt	12 (80.0 %)
Ballistic	3 (20.0 %)
Closed	8 (53.3 %)
Open	7 (46.7 %)
External fixation	6 (40.0 %)
Average time to definitive fixation (days)	4.7
Smoker	5 (33.3 %)
Former smoker	2 (13.3 %)
Non-smoker	8 (53.3 %)
Diabetes	1 (6.7 %)
Average BMI	25.4

Average varus and valgus alignment was 3.1 and 2.3 degrees, respectively (range 6.7 degrees valgus-6.0 degrees varus). Average estimated blood loss was 296 cc, (range 100–700 cc). Average procurvatum and recurvatum was 2.0 and 2.6 degrees, respectively (range 6.9 degrees procurvatum-3.8 degrees recurvatum). None of the 15 patients demonstrated intra-operative subsidence as determined by fluoroscopy during the procedure nor did the 13 patients available for follow-up on subsequent follow-up radiographs. One patient had prominence of a proximal interlocking screw that was removed in clinic under local anesthesia. Another patient had a 3 mm screw back-out on the tibial plate but was asymptomatic. There was a single superficial skin infection that resolved after a 14-day course of oral antibiotics.

## Discussion

Intra-articular proximal tibia fractures with associated metaphyseal or diaphyseal components are challenging fracture patterns to address surgically. Historically, these fractures have undergone fixation with large periarticular plates or dual plating methods but not with combined plate and nail constructs. There are some recent reports of combined plate-nails being used in AO/OTA 41C2/C3 bicondylar tibial plateau fractures with good results [10]. The study highlights this technique applied to more severe fracture patterns (AO/OTA 41C2/C3 +/− 42A-C) with similar clinical outcomes. Anecdotally, the intramedullary nail appears to have an added benefit for supporting any metaphyseal or diaphyseal comminution to prevent varus collapse and allow the selected group of patients to return to early weightbearing. More studies are needed to assess the biomechanical advantages or limitations associated with plate-nail constructs. Intramedullary nails may also support injuries with medial-sided fracture patterns using a technique that maintains the medial soft tissue envelope and periosteum that may be damaged with surface plating [10].

A combined approach may be especially useful in mobilizing the multiple-injured patient, as these fracture patterns are typically encountered in high-energy trauma with concomitant injuries. Compared to isolated nailing, the study did not find a minimum distance needed between tibial anterior cortex and proximal interlocking screws, due to sufficient reconstruction and stabilization of the proximal tibia articular surface through compression and rafting principles applied via plating. Thus, the tibial interlocking screws provided both additive fixation and rotational and length stability to the metaphyseal/diaphyseal component rather than necessity fixation for the proximal articular component*.* Combined plating and nailing impart the additional advantage of limiting further soft tissue injury or periosteal stripping associated with larger open techniques seen in dual plating methods. This benefit can be accomplished through a minimally invasive plate osteosynthesis (MIPO) technique and a suprapatellar or extended para-patellar approach, as shown in the study. Often, these complex tibia fractures are treated in a staged manner. However, there is some literature to support early definitive fixation [22]. Utilizing a MIPO technique and percutaneous nail passage may allow for early definitive fixation, as the patients in this study underwent definitive surgery an average of 5 days from the time of injury, with 8 out of 15 patients undergoing definitive surgery on hospital day 1 and the remaining 7 patients receiving definitive management later in their hospital course. MIPO techniques are particularly useful in the setting of open fractures [23].

It is at the discretion of the surgeon to identify fracture patterns that are amenable to a plate-nail construct. The study found that AO/OTA fractures 41C2/3, especially those with associated AO/OTA 42 components, are agreeable to this fixation strategy. However, some potential pitfalls of this combined approach include challenges in determining implant trajectories that do not overlap, which could lead to implant convergence, as well as variations in pre-operative planning of the implant placement sequence, which largely depend on the clinical context and surgeon preference. Regarding fractures likely unsuitable for a combined approach, patients with heavy soft tissue injury or fractures with extensive inflammation and swelling are less than ideal, given the need for additional incisions around the knee. Additionally, patients with limitations to conventional intramedullary nailing, such as anterior cortex blowouts, are not suitable for this combined approach, as anterior cortical bone provides stabilization to the tibial nail that is critical in the anterior-posterior plane. Meticulous pre-operative evaluation with special attention to the soft tissues and thorough radiographic analysis is vital for successful patient selection.

Other approaches, including infrapatellar and lateral parapatellar approaches that utilize a lateral compartment incision, can also potentially be used but were not attempted in this case series. The concern with these approaches, however, is the potential for increased incision burden and/or lack of distance between incisions. Complex proximal tibia fractures are known to be at risk of deformity; as such, semi-extended nailing can also be considered to help reduce the likelihood of introducing deformity to the joint. Additional alternative techniques for these fractures include long MIPO plating or isolated nailing; however, each has intrinsic limitations. Long MIPO plating across significant metaphyseal or diaphyseal comminution can encourage plate fatigue, bending, and typically requires dual plate constructs, while isolated nailing alone may be insufficient in plateau fixation. Regardless of approach, the goals of surgery should be focused on an anatomic reduction of the joint surface with maintenance of length, alignment, and rotation for any associated metaphyseal or diaphyseal fractures.

## Conclusion

In summary, combined proximal tibia plate and intramedullary nail for intra-articular proximal tibia fractures that demonstrate metaphyseal extension or have associated shaft fractures can be a successful technique in the armamentarium of orthopaedic trauma surgeons. The procedure, when applied to an appropriate injury and patient, may allow for an earlier return to weightbearing, without sacrificing alignment or additional soft tissue injury.

## CRediT authorship contribution statement

**Daniel Marks:** Validation, Methodology, Investigation, Formal analysis, Data curation, Conceptualization, Writing – review & editing, Writing – original draft. **Matthew Dulas:** Methodology, Investigation, Formal analysis, Data curation, Writing – review & editing, Writing – original draft. **James Dahm:** Formal analysis, Data curation, Conceptualization. **Anthony Christiano:** Supervision, Project administration, Writing – review & editing, Writing – original draft. **Jason Strelzow:** Supervision, Project administration, Conceptualization, Writing – review & editing, Writing – original draft. **Solomon Egbe:** Writing – review & editing, Writing – original draft.

## Funding sources

This research did not receive any specific grant from funding agencies in the public, commercial, or not-for-profit sectors.

## Declaration of competing interest

The authors declare the following financial interests/personal relationships which may be considered as potential competing interests: Jason Strelzow, MD reports a relationship with Acumed LLC that includes: speaking and lecture fees. Jason Strelzow, MD reports a relationship with American Society for Surgery of the Hand that includes: board membership. Jason Strelzow, MD reports a relationship with BoneSupport that includes: consulting or advisory and speaking and lecture fees. Jason Strelzow, MD reports a relationship with Journal of Bone and Joint Surgery Inc. that includes: board membership. Jason Strelzow, MD reports a relationship with Journal of Hand Surgery that includes: board membership. Jason Strelzow, MD reports a relationship with Orthopaedic Trauma Association that includes: board membership. Jason Strelzow, MD reports a relationship with OrthoXel that includes: consulting or advisory. Jason Strelzow, MD reports a relationship with Stryker that includes: consulting or advisory. If there are other authors, they declare that they have no known competing financial interests or personal relationships that could have appeared to influence the work reported in this paper.
